# Analysis of the role of PCNA-DNA contacts during clamp loading

**DOI:** 10.1186/1472-6807-10-3

**Published:** 2010-01-30

**Authors:** Randall McNally, Gregory D Bowman, Eric R Goedken, Mike O'Donnell, John Kuriyan

**Affiliations:** 1 Department of Molecular and Cell Biology, Department of Chemistry, California Institute for Quantitative Biosciences (QB3), Howard Hughes Medical Institute, University of California, Berkeley, Berkeley, CA 94720, USA; 2 Physical Biosciences Division, Lawrence Berkeley National Laboratory, Berkeley, CA 94720, USA; 3 Howard Hughes Medical Institute, Rockefeller University, 1230 York Avenue, Box 228, New York, NY 10021, USA; 4 Current address: Department of Biophysics, Johns Hopkins University, 3400 North Charles Street, Baltimore, MD 21218-2685, USA; 5 Current address: Abbott Bioresearch Center, 100 Research Drive, Worcester, MA 01605, USA

## Abstract

**Background:**

Sliding clamps, such as Proliferating Cell Nuclear Antigen (PCNA) in eukaryotes, are ring-shaped protein complexes that encircle DNA and enable highly processive DNA replication by serving as docking sites for DNA polymerases. In an ATP-dependent reaction, clamp loader complexes, such as the Replication Factor-C (RFC) complex in eukaryotes, open the clamp and load it around primer-template DNA.

**Results:**

We built a model of RFC bound to PCNA and DNA based on existing crystal structures of clamp loaders. This model suggests that DNA would enter the clamp at an angle during clamp loading, thereby interacting with positively charged residues in the center of PCNA. We show that simultaneous mutation of Lys 20, Lys 77, Arg 80, and Arg 149, which interact with DNA in the RFC-PCNA-DNA model, compromises the ability of yeast PCNA to stimulate the DNA-dependent ATPase activity of RFC when the DNA is long enough to extend through the clamp. Fluorescence anisotropy binding experiments show that the inability of the mutant clamp proteins to stimulate RFC ATPase activity is likely caused by reduction in the affinity of the RFC-PCNA complex for DNA. We obtained several crystal forms of yeast PCNA-DNA complexes, measuring X-ray diffraction data to 3.0 Å resolution for one such complex. The resulting electron density maps show that DNA is bound in a tilted orientation relative to PCNA, but makes different contacts than those implicated in clamp loading. Because of apparent partial disorder in the DNA, we restricted refinement of the DNA to a rigid body model. This result contrasts with previous analysis of a bacterial clamp bound to DNA, where the DNA was well resolved.

**Conclusion:**

Mutational analysis of PCNA suggests that positively charged residues in the center of the clamp create a binding surface that makes contact with DNA. Disruption of this positive surface, which had not previously been implicated in clamp loading function, reduces RFC ATPase activity in the presence of DNA, most likely by reducing the affinity of RFC and PCNA for DNA. The interaction of DNA is not, however, restricted to one orientation, as indicated by analysis of the PCNA-DNA co-crystals.

## Background

The faithful and efficient copying of chromosomal DNA is performed by chromosomal replicases, which generate double-stranded DNA from primed, single-stranded DNA [1,2]. These replicases achieve great speed and processivity by tethering to ring-shaped protein complexes called sliding clamps, which encircle DNA and have the ability to slide freely along the DNA strand [3,4]. The DNA polymerase subunits of these replicases maintain their association with the template strand by remaining attached to the clamp, which moves along with the polymerases during DNA synthesis. The *E. coli* replicase (DNA polymerase III) is one of the best-studied chromosomal replicases, and it has been shown that in the absence of the sliding clamp, the replicative polymerase cannot synthesize more than a few bases without falling off. In contrast, the presence of the sliding clamp allows thousands of nucleotides to be incorporated at speeds up to 1000 base pairs per second [5,6]. Similar conclusions have been reached for the replicase of T4 bacteriophage [7,8].

The general architecture of sliding clamps is conserved throughout all domains of life [9-11]. In *E. coli*, the sliding clamp is the β subunit of the pol III holoenzyme (β clamp), and the eukaryotic sliding clamp is called Proliferating Cell Nuclear Antigen (PCNA). The sliding clamps share a domain architecture that has a six-fold pseudosymmetric rotation axis running through the center of the clamp and a central pore lined with α-helices through which DNA is threaded [10,11]. The clamps have two unique faces, one of which (referred to here as the proximal face) is used for interaction with binding partners, such as the polymerases and clamp loader complexes [10-13]. The other face (referred to here as the distal face) has not been implicated in functional interactions.

**Figure 1:**
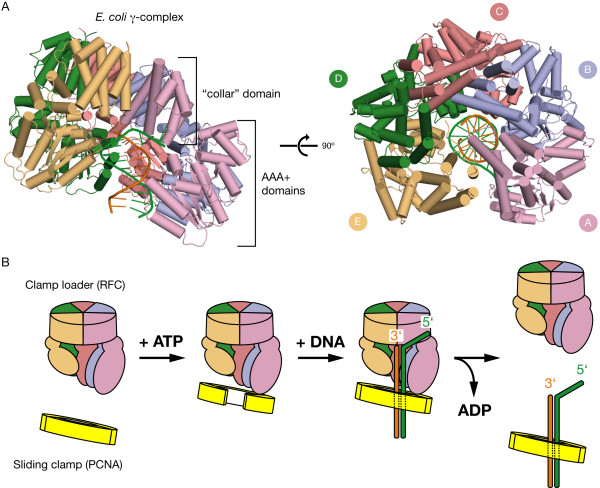
**Clamp loader structure and clamp loader cycle**. (A) The *E. coli *clamp loader, γ-complex, bound to primer-template DNA [30]. On left, the domain structure of the clamp loader subunits is noted. (B) Schematic diagram of the clamp loader cycle.

Sliding clamps from bacteria and eukaryotes share little sequence similarity and have different subunit stoichiometries. The *E. coli* β clamp has two identical subunits that each contain three similar domains (it is thus a "dimer of trimers") [10] while PCNA has three identical subunits that each contain two similar domains (a "trimer of dimers") [11]. Sliding clamps typically exist as closed rings in solution, and so they do not load onto DNA readily. The loading of clamps onto DNA is facilitated by clamp loader complexes, which are five-subunit AAA^+ ^ATPases [14,15]. Based on crystal structures, clamp loader subunits are labeled A-E (Figure 1A). Each subunit contains a AAA+ ATPase module (domains I and II) and a C-terminal ''collar'' domain (domain III) that oligomerizes the clamp loader. The ATP binding sites of these complexes are formed at the interfaces between adjacent subunits. ATP hydrolysis is triggered by an "arginine finger" supplied by a neighboring subunit to the subunit to which the ATP is bound [14-17].

To bind, open, and load the clamp on DNA, the clamp loader executes a cycle of ATP binding and hydrolysis. ATP binding allows for the formation of a stable complex between the clamp loader and an open clamp [18,19]. Upon binding primer-template DNA [20], ATP hydrolysis is stimulated, and results in the release of the clamp on DNA (Figure 1B) [21-23].

The central hole within the sliding clamp is lined with α-helices that present a number of conserved, positively charged residues [10,11], creating a surface that complements the negatively charged DNA backbone. Because a strong interaction between the inner surface of the sliding clamp and DNA might hinder movement of the polymerase, tight binding of DNA to the clamp in a specific orientation is not expected. Molecular dynamics simulations have predicted that while DNA has a preference to be oriented within the hole in PCNA at an angle of about 20° relative to the axis of PCNA, the DNA is also highly mobile and is not strictly restricted to any particular position [24].

Given the transient nature of the interaction between DNA and the clamp that is expected, the crystal structure of the *E. coli *β clamp bound to primer-template DNA was a surprise [25]. This structure showed that DNA was well localized in the center of the clamp, making an angle of 22° with the axis of the clamp. The β clamp-DNA structure also revealed that the single-stranded overhang of the DNA binds to the polymerase binding site on the proximal face of the clamp, and that mutation of residues at that interface causes deficiencies in clamp loading [25]. Thus, at least for the bacterial replicase, a specific interaction between primer-template junctions and the clamp might hold the clamp in position prior to arrival of the polymerase [25]. Two residues in the β clamp that make contact with the double-stranded region of the DNA in the structure (Arg 24 and Gln 149) were shown to be necessary for efficient clamp loading [25] and have also been implicated in the ability of the clamp loader to discriminate between a 3' or 5' primer-template junction [26]. The interaction of a sliding clamp with a tilted DNA was also observed in an electron microscopic reconstruction of DNA ligase bound to PCNA and DNA [27].

Several of the positively charged residues in the central helices of PCNA have been shown to be essential for the stimulation by PCNA of the activity of eukaryotic DNA polymerase δ [28], as well as resulting in deficient mismatch repair when mutated [29]. In contrast, none of the residues in the center of PCNA have been implicated so far in clamp loading. The residues in the β clamp-DNA structure (Arg 24 and Gln 149) that make contact with double-stranded DNA and have been implicated in clamp loading reside in loops on the proximal face of the clamp [25] rather than in the central hole.

This work presents mutational analysis that shows that interactions between DNA and several positively charged residues in the center of *S. cerevisiae *PCNA play a role in the clamp loading process. In addition, we present a model of PCNA bound to DNA that is derived from X-ray diffraction data, which shows interactions between DNA and an additional set of positively charged residues in the center of PCNA.

## Results & Discussion

### A Model for PCNA-DNA Interaction During Clamp Loading

To determine if PCNA-DNA interactions have relevance for clamp loading, a RFC-PCNA-DNA model was generated to visualize the path of the DNA through PCNA during the clamp loading process. In order to construct this RFC-PCNA-DNA model, we combined features of two different crystal structures, the *E. coli *clamp loader (γ-complex) bound to primer-template DNA (Figure 1A) [30], and the *S. cerevisiae *clamp loader (RFC) bound to closed PCNA [31]. We aligned the RFC-PCNA structure onto the γ-complex structure using the B subunits of the two complexes. This alignment allows us to transfer bound DNA from the γ-complex structure to that of RFC, and is justified because of the similarity between the general organization of the ATPase domains in the two structures [30]. Each ATP-coordinating subunit-subunit transition in the γ-complex-DNA structure maintains a rotation and translation that aligns along the axis of the bound DNA, and this transition is maintained roughly in the RFC-PCNA structure.

**Figure 2:**
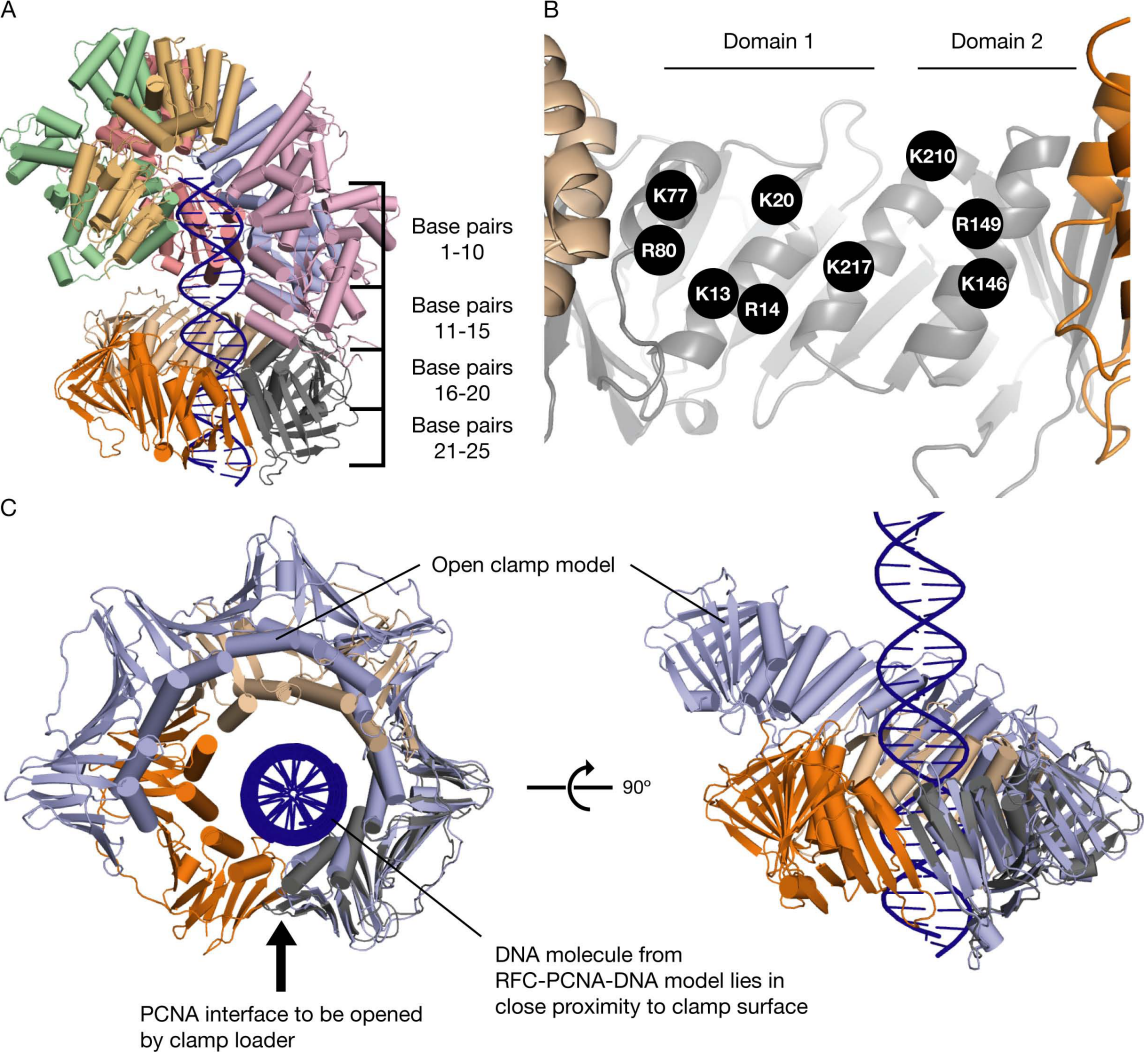
**Model of RFC bound to PCNA and DNA**. (A) The RFC-PCNA-DNA model, which depicts the path of DNA bound to the clamp loader and through the clamp, combines features of a DNA-bound γ-complex structure [30] and an RFC-PCNA structure [31]. (B) The α-helices lining the hole of PCNA, with the positions of the nine arginines and lysines that reside on them indicated. (C) Top and side views of the positions of the DNA molecule from the RFC-PCNA-DNA model relative to PCNA, with a model of PCNA opened by RFC overlaid upon the closed PCNA. The overlay of the open clamp shows that much of the PCNA-DNA interaction remains in place when the clamp is opened and pulled out of axis.

The DNA model that was transferred to RFC from the γ-complex was then extended through the clamp by aligning a long ideal B-form DNA model to the γ-complex-bound DNA. The resulting RFC-PCNA-DNA model depicts RFC bound to PCNA and B-form DNA (Figure 2A). The DNA does not pass through the center of the clamp, but close to one side, at a proximity that allows interaction with each of the positively charged residues on the helices lining the center of the clamp (Figures 2C &6D).

Because the PCNA in the RFC-PCNA-DNA model is closed, the step in the clamp loader cycle it represents would likely be after the clamp has been loaded but before release by the clamp loader, or perhaps a transient closed state sampled by the clamp during the process of recognizing DNA. To determine if an open PCNA during clamp loading could contact DNA in a manner analogous to that modeled for a closed clamp, a model of an open PCNA was aligned onto the PCNA in the RFC-PCNA-DNA model. The open PCNA model was generated using molecular dynamics (MD) simulations in an earlier work [32] and was aligned on the PCNA subunit that contacts the A subunit of RFC. This results in the open clamp following the spiral formed by the RFC subunits [32,33]. The alignment shows that while part of the open PCNA is pulled away from the DNA in an out-of-plane manner, interactions with RFC allow the PCNA subunit that makes much of the contact (gray subunit, Figure 2C) with DNA to remain in the same position whether PCNA is open or closed.

### ATPase Assays Show that Positively Charged Residues in the Center of PCNA Participate in Clamp Loading

To determine if the center of the clamp interacts with DNA during clamp loading as suggested by the RFC-PCNA-DNA model, all nine of the arginine and lysine residues that reside on the helices lining the center of *S. cerevisiae *PCNA (Figure 2B) were mutated individually and their effect on DNA-dependent ATPase stimulation of RFC was assayed. Because ATP hydrolysis occurs as the clamp is released onto DNA, ATPase activity serves as a useful readout of clamp loading activity.

**Figure 3:**
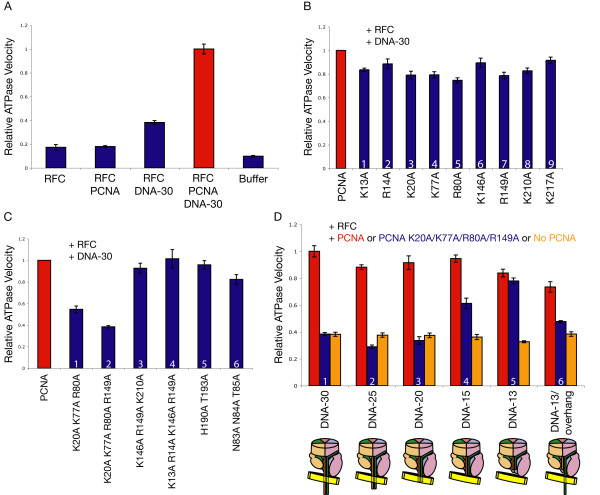
**Stimulation of DNA-dependent ATPase activity of RFC by mutant clamps**. (A-D) Results of enzyme-coupled ATPase assay (each reaction contained 50 nM RFC, 100 nM PCNA, 100 nM DNA, and 0.5 μM ATP; see Methods). The ATPase rate of each reaction is displayed relative to the ATPase rate of RFC in the presence of PCNA and DNA-30, which is scaled to a value of one. Error bars represent standard deviation of multiple trials. DNA-30 has 30 base pairs in the duplex region and a 10-base 5' overhang on the primer strand; DNA-25 has 25 base pairs in the duplex region, etc. DNA-13/overhang is identical to DNA-13 but contains a 17-base 3' overhang on the template strand. (A) Wild-type PCNA stimulates RFC ATPase activity in the presence of DNA. (B) The point mutations in PCNA that result in the largest effects on ATPase activity in the presence of DNA are R80A and R149A, which have deficiencies of only 20-25% relative to wild-type PCNA. (C) Simultaneous mutation of PCNA residues K20A, K77A, R80A, and R149A results in an inability of PCNA to stimulate DNA-dependent ATPase activity. (D) PCNA K20A/K77A/R80A/R149A is deficient in stimulating ATPase activity when the primer-template DNA constructs present are long enough to extend through the clamp during loading, but its activity approaches that of wild-type PCNA in the presence of short DNA that cannot reach into the clamp.

The ATPase activity of RFC was measured using an enzyme-coupled reaction in which the production of ADP is coupled to the depletion of NADH by pyruvate kinase and lactate dehydrogenase; the rate of NADH depletion is monitored by UV spectrometry [34]. RFC alone and RFC in the presence of PCNA have a low basal level of ATPase activity; the rate of this activity is increased in the presence of DNA. The maximal rate of ATP hydrolysis is achieved with the addition of PCNA and primer-template DNA together (Figure 3A).

Using the RFC-PCNA-DNA ATPase level as a benchmark, the ATPase activity of RFC with DNA and the PCNA mutants was measured (Figure 3B). The DNA construct used in the assay (called DNA-30) has a 30-base pair duplex region with a 10-base 5' overhang. Each of the PCNA mutants showed either a modest decrease in DNA-dependent ATPase stimulation of RFC or no significant effect. The largest effects were observed for PCNA R80A and PCNA R149A, but these corresponded to only a 20-25% reduction in ATPase activity compared to wild-type PCNA. Indeed, a previous study did not detect an effect on DNA-dependent RFC ATPase activity from individually mutating each of the positive residues in the center of human PCNA [28]. The minimal effects of these mutations might reflect the fact that each point mutation removes only one-ninth of the positively charged residues in the center of PCNA.

Because the effects of single mutations were so small, it was necessary to mutate several positive residues in the center of PCNA in combination to produce a more robust effect. A number of combinations of triple mutants and quadruple mutants were studied. The triple mutant PCNA K20A/K77A/R80A was generated to test the effect of mutating residues on the α-helices of domain 1 (Figure 2B) of PCNA, while PCNA K146A/R149A/K210A tested the effects of mutating residues of domain 2. PCNA K20A/K77A/R80A/R149A combines the point mutations that individually had the four largest effects on DNA-dependent RFC ATPase activity as measured above.

The results (Figure 3C) indicate that the domain 2 triple mutant, PCNA K146A/R149A/K210A, shows no decrease in RFC ATPase activity in the presence of DNA. In contrast, a significant decrease in ATPase activity was seen for the domain 1 triple mutant PCNA K20A/K77A/R80A and the quadruple mutant PCNA K20A/K77A/R80A/R149A (which are the same except for an additional mutation in the latter). Strikingly, the ATPase activity with PCNA K20A/K77A/R80A/R149A present was the same as that observed in the absence of PCNA.

The drastic effects on PCNA-stimulated and DNA-dependent RFC ATPase activity for the PCNA K20A/K77A/R80A and PCNA K20A/K77A/R80A/R149A mutants suggest that interaction of the mutated residues with DNA plays an important role during the loading process. To verify whether contact with DNA by these residues in the center of PCNA is a necessary step for the stimulation of RFC ATPase activity, ATPase assays were performed with PCNA K20A/K77A/R80A/R149A and DNA constructs of various lengths, the shortest of which are too short to extend beyond the RFC complex and into the clamp. The RFC-PCNA-DNA model predicts that 25 base pairs in the duplex region of DNA would extend completely through the clamp, that 20 base pairs would extend partially into the clamp, and that 15 base pairs would terminate near the opening of the clamp (Figure 2A). DNA constructs shorter than 15 base pairs are predicted to be too short to contact residues in the center of PCNA when bound to RFC. Each DNA construct used contained 10-base 5' overhangs and was named according to the length of its duplex region (DNA-25 has 25 base pairs in the duplex region, etc.).

The results show that the length of the DNA constructs did not affect their ability to stimulate RFC ATPase activity when no PCNA is present (Figure 3D, orange columns 1-5). Also, for each of the DNA constructs, wild-type PCNA stimulated RFC ATPase activity to approximately the same level (Figure 3D, red columns 1-5). In the presence of DNA constructs that were predicted to enter or pass through the clamp during loading (DNA-30, DNA-25, and DNA-20), PCNA K20A/K77A/R80A/R149A failed to stimulate RFC ATPase activity (Figure 3D, blue columns 1-3). In the presence of the DNA construct predicted to extend near the opening of the clamp, DNA-15, the mutant PCNA stimulated ATPase activity to an intermediate level (Figure 3D, blue column 4). Finally, for the shortest DNA construct DNA-13, the mutant PCNA approaches wild-type PCNA in ability to stimulate RFC ATPase activity (Figure 3D, blue column 5). The DNA length dependence of these results suggests that the lack of contact between certain positively charged residues in the center of PCNA and DNA passing through the clamp negatively affects the clamp loading process. The ability of the mutant PCNA to stimulate RFC in the presence of DNA-13 indicates that the mutations did not impair the stability of the clamp.

To examine whether single-stranded DNA passing through the clamp might also affect clamp loading, a DNA construct that had a 13-base duplex region with an overhanging 17-base 3' strand (DNA-13/overhang) was tested. PCNA K20A/K77A/R80A/R149A showed a large deficiency in stimulating RFC ATPase activity in the presence of this DNA construct, in marked contrast to the full activation seen in the presence of DNA-13, which is a similar DNA construct but lacks the 3' overhang (Figure 3D, blue columns 5 & 6). This suggests that interaction between single-stranded DNA and the clamp could take place during clamp loading.

PCNA K20A/K77A/R80A/R149A is not deficient in stimulating RFC ATPase activity in the presence of a short DNA construct that does not extend through the center of PCNA (Figure 3D). This suggests that while residues at the center of PCNA are not necessary to stabilize a conformation of RFC capable of ATPase activation and DNA binding when the DNA is too short to contact them, they are necessary when the DNA is long enough to reach them. The eukaryotic primase, pol α, generates a RNA/DNA primer strand longer than 30 bases, which is easily long enough to thread through the clamp during loading, emphasizing the importance of the interactions with PCNA [1,35].

### Mutation of Positive Residues in the Center of PCNA Results in Deficient RFC-PCNA Binding to DNA

There are two possible reasons that the loss of PCNA residues Lys 20, Lys 77, Arg 80, and Arg 149 interrupts the ATPase activity of RFC in the presence of DNA. First, the PCNA-DNA interaction may become unfavorable, which would destabilize the position of the DNA within the RFC-PCNA-DNA complex or even disrupt formation of the ternary complex itself. Second, following formation of the RFC-PCNA-DNA complex, an unfavorable interaction between PCNA and DNA may hinder clamp closing and release of the clamp by RFC, interfering with the typical RFC ATPase cycle. Fluorescence anisotropy binding experiments were performed to measure the affinity of RFC for TAMRA-labeled DNA in the presence of wild-type PCNA or PCNA K20A/K77A/R80A/R149A. RFC was titrated into a mixture of primer-template DNA labeled with a TAMRA fluorophore on the 5' end of the template strand in the presence of PCNA (or mutant) and the non-hydrolyzable ATP analog, ATP-γ-S.

**Figure 4:**
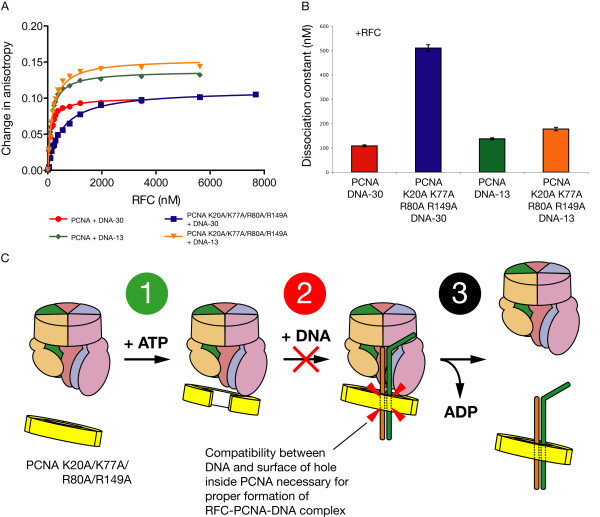
**Affinity of RFC and mutant PCNA for DNA as measured by fluorescence anisotropy**. (A) Fluorescence anisotropy binding curves for RFC titrated into a mixture of 1 μM PCNA (or PCNA K20A/K77A/R80A/R149A) and 100 nM TAMRA-labeled DNA-30 (30 base pairs in the duplex region) or DNA-13 (13 base pairs in the duplex region) in the presence of 1 mM ATP-γ-S. Curves represent best fit to a one-site binding equation (see Methods). (B) Bar graph presenting dissociation constants of binding data from Figure 4A. Error bars represent standard error of the curve fit. (C) The clamp loader cycle is broken into three parts; fluorescence anisotropy binding data suggests that step 2 of the cycle, the formation of the RFC-PCNA-DNA ternary complex, is hindered by PCNA K20A/K77A/R80A/R149A through the loss of compatibility between DNA and the surface of the clamp's hole.

With no PCNA present, RFC has a weak affinity for DNA; in our experiments, RFC and DNA alone failed to produce a saturating binding curve (data not shown). With PCNA present, however, RFC was bound to labeled DNA-30 with a dissociation constant (K_d_) close to 100 nM. In contrast, the K_d _of RFC for DNA-30 in the presence of PCNA K20A/K77A/R80A/R149A was nearly 5 times weaker (Figures 4A &4B). When DNA-13 is used, which is too short to extend through the clamp, the affinity of RFC for the DNA changes little whether the PCNA present is wild-type or the mutant (Figures 4A &4B).

The anisotropy binding data allows us to determine which step of the three-part clamp loader cycle is affected by the mutant clamp in the presence of a long DNA construct. In step 1 of the cycle, ATP-loaded RFC binds and opens PCNA; in step 2, RFC and PCNA bind to DNA; and in step 3, ATP is hydrolyzed and the clamp is released around the DNA (Figure 4C). The mutations do not affect step 1; because the mutant PCNA performed at wild-type level with the shortest DNA in both ATPase stimulation and DNA binding affinity, it is clear that the mutant clamp is capable of binding RFC. However, the anisotropy binding data using the long DNA suggests that the formation of the RFC-PCNA-DNA ternary complex in step 2 is hindered when contact between DNA and the surface of the PCNA hole is rendered incompatible by mutations. This effect may account for much of the deficiency in ATPase activity when the mutant PCNA is used for longer DNA constructs. Finally, it is possible, perhaps likely, that step 3 is also affected by the mutant clamp in the presence of long DNA. It is reasonable to expect that loss of DNA-contacting residues would hinder closing of the clamp and its release around DNA.

### A Model for PCNA Bound to DNA Derived from X-ray Diffraction Data

The ATPase and anisotropy binding experiments presented above show that DNA interacts with the center of PCNA during clamp loading. To structurally characterize interactions between the center of PCNA and DNA, we set out to solve the X-ray crystal structure of a PCNA-DNA complex. A key to the successful determination of the structure of the β clamp-DNA complex [25] was the use of DNA labeled with a Cy5 chromophore that colored the DNA-containing crystals blue. Our crystallization of *S. cerevisiae *PCNA in complex with DNA used a similar approach. Blue crystals of wild-type PCNA were obtained readily, in the presence of primer-template DNA of varying lengths labeled with a Cy5 molecule on the 5' end of the primer strand. Electron density maps calculated after placing PCNA in the unit cell by molecular replacement showed no features corresponding to DNA. As for crystals of *S. cerevisiae *PCNA alone [11], these newly obtained blue crystals belong to a cubic space group that features a crystallographic three-fold rotation axis running through the center of PCNA. This results in three-fold rotational averaging of electron density in the center of the PCNA ring, thereby obscuring features arising due to DNA.

**Figure 5:**
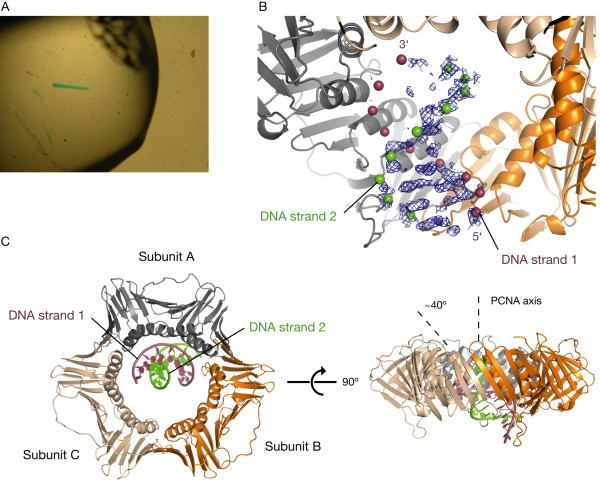
**X-ray derived model of PCNA bound to DNA**. (A) Crystals of single-chain PCNA-DNA. The DNA is labeled on the 5' end of the primer strand with a Cy5 chromophore. (B) Unbiased electron density for the DNA produced from single-chain PCNA-DNA crystal, calculated before DNA was placed into the model. 2F_o_-F_c _map is displayed at a contour level of 0.7σ. Positions of the DNA phosphates in the final model are shown as spheres. (C) PCNA-DNA model derived from X-ray diffraction data. The DNA makes a ~40° angle with the central axis of PCNA.

To overcome this problem, we generated a single-chain construct of PCNA in which three PCNA monomers are fused together by two 11-residue linkers (specified in Methods). We screened for crystals using protein derived from this construct and Cy5-labeled primer-template DNA constructs of varying lengths, and obtained crystals of several forms that incorporated the labeled DNA and appeared bright blue (Figure 5A).

We measured X-ray data to 3.0-3.5 Å resolution for several of these crystals. Data from most of these crystals did not reveal electron density features that could be attributed unambiguously to a bound DNA molecule. For a minority of crystals, however, electron density maps calculated using PCNA placed by molecular replacement revealed features that are clearly recognizable as being due to DNA. The space group for these single-chain PCNA-DNA crystals is P2_1_2_1_2_1_, with one trimer of PCNA in the asymmetric unit. We used one of these datasets for the analysis presented here, derived from a crystal that incorporated a 10-base pair DNA construct with a four-base 5' overhang on one end. Electron density maps calculated using this dataset reveal weak features that could be interpreted in terms of five base pairs for which stacked DNA bases and parts of the phosphate backbone could be seen (Figure 5B). Based on this weak density, we placed a 10-base pair B-form DNA in the center of the PCNA ring at a tilt of ~40° with respect to the rotation axis of PCNA (Figure 5C). Refinement of coordinates and temperature (B) factors for individual atoms in this PCNA-DNA model resulted in very high temperature factors for the atoms in the DNA (in the range of about 125-450 Å^2^). This indicates that the DNA is partially disordered or has low occupancy, and the resolution of the dataset does not permit reliable refinement of individual atomic positions for the DNA. Attempts to verify the placement of the DNA by anomalous phasing of bromodeoxyuridine (BrdU) incorporated into the DNA strand were unsuccessful, possibly because of disorder in the DNA. As a result, we present PCNA-DNA not as a definitive crystal structure but rather as an X-ray derived model. We have deposited the coordinates of the PCNA-DNA model and experimental structure factors in the RCSB Protein Data Bank (ID code 3K4X). Table 1 presents crystallographic statistics for the refined PCNA-DNA model.

**Table 1:** Crystallographic Data and Refinement Statistics

Data Collection	
Beamline	ALS 8.2.2
Wavelength (Å)	1.00
Space group	P2_1_2_1_2_1_
Cell (Å)	83.9 93.7 136.9
Resolution (Å)	50.00-3.00 (3.11-3.00)
R_sym _(%)	14.8 (57.4)
I/σ	10.7 (1.9)
Completeness (%)	97.9 (88.2)
Redundancy	5.2 (4.0)

**Refinement**	

Reflections	22002
Test set	1121
Number of atoms	6761
Protein	6354
Nucleic acid	407
R_factor _(%)	22.3
R_free _(%)	28.0
r.m.s. deviation, bond lengths (Å)	.003
r.m.s. deviation, bond angles (°)	.667

* Outer resolution shell and statistics for that shell are in parentheses

In an attempt to crystallographically trap an open clamp, the single-chain PCNA contained mutations R110S and Y114S in the first subunit [36,37]. The PCNA in the model is very well resolved and, despite these mutations, is closed and does not significantly deviate from wild-type PCNA. The inter-subunit linkers engineered into the single-chain PCNA were not resolved in the electron density, making it difficult to determine where the DNA lies in relation to the one unconstrained PCNA interface. The four-base DNA overhang was also not visible in the experimental maps. As a result, it is unknown which end of the DNA in the model is blunt and which is the primer-template junction. Because the strongest DNA density lies towards the distal face of PCNA (the face that does not bind polymerases) and density corresponding to the overhang is not visible there, the identities of the DNA bases are assigned as if this is the blunt end. The DNA strand labeled as the primer strand in the model will be referred to as DNA strand 1 and the template strand will be referred to as DNA strand 2 (Figures 5B &5C).

**Figure 6:**
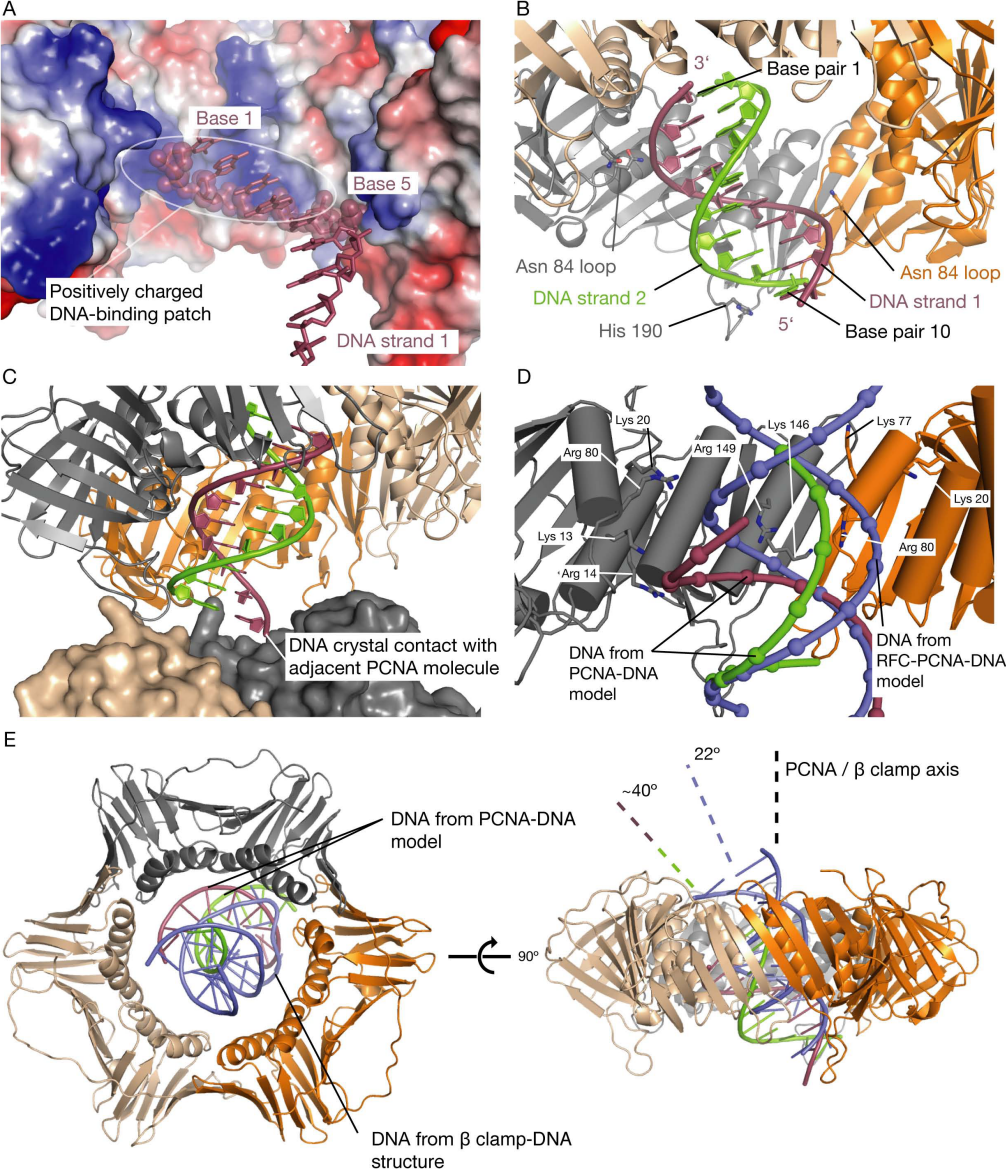
**PCNA-DNA contacts in the X-ray derived PCNA-DNA model**. (A) Electrostatic surface representation of the center of PCNA; positive electrostatic surfaces are colored blue, negative surfaces are red, and neutral surfaces are white. Note the interactions made between the negatively charged backbone of DNA strand 1 (depicted as spheres) and the positively charged patch located in the center of PCNA formed by residues Lys 13, Arg 14, Lys 146, and Arg 149. The PCNA subunit in the foreground is removed for clarity. (B) Contacts between DNA and the His 190 loop and Asn 84 loops are indicated. (C) The PCNA-DNA model and an adjacent PCNA molecule in the crystal lattice (the adjacent PCNA molecule is rendered as a surface). The DNA end protruding from the distal face of the clamp forms a crystal contact. (D) Close-up view of the positions of the DNA molecules from the RFC-PCNA-DNA model and the PCNA-DNA model relative to PCNA. The angles of the DNA molecules in the RFC-PCNA-DNA model and the PCNA-DNA model differ by ~26°. Note the positions of residues identified to interact with DNA from the RFC ATPase assay and the PCNA-DNA model. DNA phosphates are shown as spheres. A PCNA subunit is removed from the foreground for clarity. (E) Overlay of PCNA-DNA model and β clamp-DNA structure [25], aligned on their respective clamps. The β clamp is removed for clarity. On the right, the angles of the two DNA molecules are compared relative to the rotation axis of the clamp to which they are bound.

Inspection of the position of the partially disordered DNA in our model reveals potential interactions between DNA and the center of PCNA. The most extensive type of PCNA-DNA interaction occurs on DNA strand 1; a number of positively charged residues that reside on the helices lining the center of PCNA, especially Lys 13, Arg 14, Lys 146, and Arg 149 of subunit A and Lys 146 and Arg 149 of subunit C, create a surface that complements the negatively charged phosphate oxygens of bases 1 to 5 (see numbering, Figures 6A &6D). Other interactions occur at loops that protrude from PCNA and make contact with the DNA backbone. The first of these loops extends from the distal face of PCNA and presents His 190 of subunit A to the phosphate group of base 10 of DNA strand 2 (Figure 6B). A second loop, which extends from the lower portion of the central pore of PCNA and presents the sidechains of Asn 83 and Asn 84 towards the DNA, makes contact with DNA strand 1 from two different subunits; from subunit A to the phosphate group of base 2, and from subunit B to the phosphate groups of bases 6 and 7 (Figure 6B). A crystal contact between the DNA end protruding from the distal face of PCNA and an adjacent PCNA molecule in the crystal lattice also contributes to holding the DNA in place (Figure 6C).

To examine whether the PCNA-DNA interactions in the X-ray model participate in clamp loading, residues from the PCNA-DNA model that contact DNA strand 1 were simultaneously mutated (PCNA K13A/R14A/K146A/R149A) and assayed for ability to stimulate RFC ATPase activity in the presence of DNA. This mutant had no effect on DNA-dependent RFC ATPase activity (Figure 3C, blue column 4). The effects of mutations in the two proposed DNA-contacting loops of PCNA, the His 190 loop and the Asn 84 loop, were also tested (Figure 3C, blue columns 5 & 6). Mutation of the two polar residues together in the His 190 loop, His 190 and Thr 193, to alanine had no significant effect on DNA-dependent ATPase activity of RFC. For the Asn 84 loop, a triple mutant was produced in which the three polar residues Asn 83, Asn 84, and Thr 85 were mutated to alanine simultaneously; this mutant also displayed only a small decrease in the ability to stimulate DNA-dependent RFC ATPase activity.

The positively charged residues in the α-helices lining the hole of PCNA that interact with DNA in our PCNA-DNA model (Lys 13, Arg 14, Lys 146, and Arg 149) do not affect RFC ATPase activity when mutated, and largely do not overlap with residues that were found to have the greatest effect on ATPase activity during loading (Lys 20, Lys 77, Arg 80, and Arg 149). In addition, the DNA angle relative to PCNA in the PCNA-DNA model is ~26° greater than that in the RFC-PCNA-DNA model (Figure 6D), and the DNA in the PCNA-DNA model would collide with PCNA if it was lengthened towards the proximal face. Thus, the DNA angle seen in the PCNA-DNA model likely does not occur during clamp loading. However, it may be used for functions that are yet to be identified, and raises the possibility that PCNA presents different sets of positively charged residues to produce tilted PCNA-DNA interactions of various angles for different functions.

Our PCNA-DNA model adds to a growing body of evidence that DNA has a propensity to lie in the center of the clamp at an angle [24,25,27]. To compare the orientations of DNA bound to the β clamp and to PCNA, the β clamp-DNA structure and the PCNA-DNA model were aligned on the clamps. The overlay demonstrates the sharper ~40° tilt angle of the DNA in the PCNA-DNA model compared to the 22° tilt of the DNA bound to the β clamp (Figure 6E). In both structures, the tilt of the DNA results in interactions between DNA and the α-helices that line the central holes in the clamps [25]. Thus, the observed tilts of the DNA bound to both the β clamp and to PCNA appear to be partly a consequence of the optimization of contact between the negatively charged DNA backbone and the positively charged center of the clamp.

Another PCNA-DNA interaction has been reported in which the DNA adopts a tilt inside the ring; a single-particle EM reconstruction of *P. furiosis *DNA ligase in complex with PCNA and DNA displays a DNA molecule threaded through the clamp at an angle [27]. Docking of our PCNA-DNA model into the ligase-PCNA-DNA volume shows that the angle formed by the DNA with the PCNA axis in the X-ray derived model is greater than that seen in the EM volume.

## Conclusion

X-ray crystallography has provided snapshots of several steps of the clamp loader cycle, including the apo-γ-complex [38], nucleotide-bound γ-complex [39], RFC bound to closed PCNA [31], and DNA-bound γ-complex [30]. These structures, along with other biochemical studies, have established that loops facing the interior of the clamp loader recognize DNA by presenting conserved positively charged and polar residues that are positioned to track the negatively charged phosphate backbone of a bound DNA molecule [31,40,41], and that DNA binding helps create a geometry of clamp loader subunits that is activated for ATPase catalysis [30]. There is presently no high-resolution structure depicting a clamp loader-clamp-DNA ternary complex, and so less is understood about the role of DNA binding to the clamp during loading. A DNA-binding role during loading for residues located near the proximal face of the β clamp has been established [25]; in this work, however, an additional set of residues is proposed to function during clamp loading. Mutational analysis suggests that positively charged residues in the center of PCNA present a binding surface to DNA that is used during clamp loading, expanding the role of the clamp during loading beyond what is currently understood.

Mutation of Lys 20, Lys 77, Arg 80, and Arg 149 in combination had a large effect on RFC ATPase activity while triple and quadruple mutation of other positively charged residues in the center of PCNA did not. Interestingly, the contacts observed in the PCNA-DNA model derived from crystallographic data do not appear to play a role in the clamp loading process. Thus, as predicted previously by molecular dynamics simulations [24], the interaction of DNA with PCNA is relatively unrestricted, with a tilted orientation of the DNA being preferred because it maximizes electrostatic complementarity with the clamp. That one combination of mutations showed effects while others did not suggests that the placement of the mutated residues relative to the DNA passing through the clamp, and not simply their charge, is a significant factor in affecting clamp loading. Further structural studies are necessary to elucidate the exact path of the DNA through the clamp during loading and which residues of the clamp it interacts with most closely.

## Methods

### Protein Purification and Expression

*S. cerevisiae *PCNA was cloned into a modified pET-28 vector (Novagen) so that it was preceded by an N-terminal 6-His tag cleavable by PreScission™ protease. *E. coli *BL21(DE3) cells were transformed with the vector and grown at 37°C to an optical density of about 1.00, and the protein was expressed at 18°C for 12-16 hours after induction with the addition of 1 mM IPTG. The harvested cells were resuspended in Buffer A (30 mM Hepes pH 7.6, 20 mM imidazole, 500 mM NaCl, 10% glycerol, and 5 mM β-mercaptoethanol). The cells were lysed by several passes through a French pressure cell (EmulsiFlex-C5; Avestin) and then centrifuged at 40,000 rpm for 40 minutes to remove cellular debris. After the lysate was filtered through a 0.45 μm filter disk (Nalgene), it was applied to a 5 mL HisTrap FF column (GE Healthcare) and eluted with a step of 50% Buffer B (same as Buffer A but with 500 mM imidazole). The selected fractions were dialyzed against Buffer A while incubating with PreScission™ protease, and again applied to a 5 mL HisTrap FF column. The selected fractions were dialyzed against Buffer C (30 mM Tris pH 7.6, 50 mM NaCl, 10% glycerol, 2 mM DTT) and applied to a 5 mL HiTrap Q HP column (GE Healthcare) and eluted with a gradient of 0 to 50% Buffer D (same as Buffer C but with 2 M NaCl) over 20 column volumes. The selected fractions were dialyzed against Buffer E (30 mM Tris pH 7.5, 100 mM NaCl, 2 mM DTT), concentrated, and stored at -80°C.

The single-chain PCNA was cloned into the same modified pET-28 vector and was preceded by an N-terminal 6-His tag cleavable by PreScission™ protease. The C-terminal glutamate residues of the first and second PCNA subunits in the chain were eliminated, as were the N-terminal methionine residues of the second and third subunits. The linker sequence between the first and second subunits was GSNSQSNGSGA; the linker sequence between the second and third subunits was GSNSQASNSGA. The single-chain PCNA contained mutations R110S and Y114S in the first subunit [36,37]. Single-chain PCNA was expressed and purified in an identical manner to that of wild-type PCNA. PCNA mutants were expressed and purified as wild-type. *S. cerevisiae *RFC was expressed and purified as described elsewhere [31] except that after the last purification step, the sample was buffer exchanged into 30 mM Hepes pH 7.6, 200 mM NaCl, 2 mM DTT, 1 mM ATP, and 10 mM MgCl_2_, then concentrated and stored at -80°C.

### Preparation of PCNA-DNA Crystals

For crystallization of PCNA-DNA, single-chain PCNA was mixed with DNA at a 1.5 molar excess of DNA, producing a final PCNA concentration of 15 mg/mL. Crystals were grown using the hanging drop vapor diffusion method; an equal volume of well solution (100 mM Na acetate pH 4.6, 100 mM NaCl, 14% PEG 4K) was added to the PCNA-DNA mixture and equilibrated with well solution at 20°C. The oligonucleotide used in crystallization was purchased from Integrated DNA Technologies; the primer strand sequence was 5'-Cy5-CCCATCGTAT-3'. The template strand sequence was 5'-TTTTATACGATGGG-3'. The DNA was annealed by heating to >95°C and slowly cooling to room temperature. For cryoprotection, crystals were equilibrated in 100 mM Na acetate, 20% PEG 4K, 100 mM NaCl, 20% glycerol, and 200 mM DNA before looping and flash-freezing in liquid nitrogen.

### X-ray Data Collection and Refinement

X-ray data were collected on beamlines 8.2.2 and 8.2.1 (Advanced Light Source, Berkeley, CA). Reflections were processed and scaled using HKL2000 [42]. Molecular replacement solutions using *S. cerevisiae* PCNA as a search model (RCSB Protein Data Bank code 1PLQ [11]) were obtained using Phaser [43]. Structure refinement was performed using PHENIX [44] and model building was carried out using Coot [45]. The DNA was refined as a rigid body, and the positions of the individual atoms within the DNA were not refined. The atomic displacement parameters of the DNA were refined with the DNA defined as one rigid (TLS) group. The inter-subunit linkers engineered into the single-chain PCNA were disordered, and not included in the final model. It was not clear from the experimental maps which PCNA subunit contained the mutated residues R110A and Y114A, and thus these mutations were also not included in the model. The atomic coordinates and structure factors have been deposited in the Protein Data Bank (3K4X).

### ATPase Assays

ATPase activity was measured by continuously monitoring the pyruvate kinase and lactate dehydrogenase-coupled oxidation of NADH at a wavelength of 340 nm [34]. With a reaction volume of 150 μL, 0.05 μM RFC, 0.10 μM PCNA or PCNA mutant, and 0.10 μM DNA were mixed with 30 mM Tris pH 7.5, 100 mM NaCl, 0.5 mM ATP, 1 mM phosphoenolpyruvate, 0.2 mg/mL NADH, 74 units/mL pyruvate kinase, 104 units/mL lactate dehydrogenase, and 10 mM MgCl_2_. The reaction was measured at 30°C, and reaction velocity was linearly fit to the slope of data recorded over a time period of 100-500 seconds. ATPase data was collected using a Molecular Devices SpectraMax Plus microtiter plate reader. The DNA-30 oligonucleotide had a double-stranded region 30 base pairs long and a 10-base pair overhang with a 3' recessed end. The template strand sequence was 5'-GAGCATTCAAGGACTTAGGTCTGTATTTATCTACCCACAA-3'; the primer strand sequence was 5'-TTGTGGGTAGATAAATACAGACCTAAGTCC-3'. The shorter DNA constructs had an identical sequence to DNA-30 but were truncated from the blunt end. The DNA was annealed by heating to >95°C and slowly cooling to room temperature. DNA constructs were obtained from Integrated DNA Technologies.

### Fluorescence Anisotropy Binding Measurements

RFC was titrated into a reaction mixture of 30 mM Tris pH 7.5, 200 mM NaCl, 2 mM DTT, 1 mM ATP-γ-S, 10 mM MgCl_2_, and 100 nM DNA-30 or DNA-13 labeled with a TAMRA fluorophore on the 5' overhanging end (obtained from Integrated DNA Technologies). The reactions contained 1 μM PCNA or PCNA K20A/K77A/R80A/R149A. Anisotropy change upon addition of RFC was noted with an excitation wavelength of 550 nm and an emission wavelength of 580 nm using a Spex Jobin Yvon FluoroMax-3 fluorimeter with a Peltier-temperature controlled sample chamber at 25°C and Glan-Thompson calcite-prism polarizers. Data was fit using Prizm 5.0 (GraphPad Software) to the one-site binding equation Y = B_max _X/(K_d _+ X), where X is the RFC concentration, Y is the change in anisotropy, and B_max _is the maximum change in anisotropy.

## Authors' contributions

RM designed and performed research, analyzed data, and drafted the manuscript. GDB designed and performed research. ERG designed and performed research. MO'D designed research and analyzed data. JK designed research, analyzed data, and drafted the manuscript. All authors read and approved the final manuscript.
